# Kupffer Cell-Derived TNF-*α* Triggers the Apoptosis of Hepatic Stellate Cells through TNF-R1/Caspase 8 due to ER Stress

**DOI:** 10.1155/2020/8035671

**Published:** 2020-08-02

**Authors:** Wei-min Wang, Xue-song Xu, Chun-mu Miao

**Affiliations:** Department of Hepatobiliary Surgery, Second Affiliated Hospital, Chongqing Medical University, Chongqing 400010, China

## Abstract

**Purpose:**

To investigate the roles of ER stress in Kupffer cells (KCs) and KC-derived TNF-*α* in the apoptosis of hepatic stellate cells (HSCs).

**Methods:**

A rat model of liver fibrosis was established. Liver and blood serum samples were collected. Liver function assays, Masson staining, Sirius Red staining, ELISAs, and TUNEL and immunohistochemical staining were performed. Liver function, liver fibrosis, KC phenotype, inflammatory factors, and number of active HSCs were investigated. KCs were isolated, treated with tunicamycin, and then, cocultured with primary hepatic stellate cells. ELISAs, immunofluorescence staining, flow cytometry, and Western blotting were performed. KC phenotype, inflammatory factors, HSC apoptosis, and TNF-R1/caspase 8 pathway activity were examined.

**Result:**

s. ER stress in KCs reduced the levels of liver function markers, reduced the degree of liver fibrosis, and increased the number of KCs with the M1 phenotype and the expression of TNF-*α*. The increase in KC-derived TNF-*α* reduced the number of active HSCs and increased the activity of TNF-R1/caspase 8. Furthermore, ER stress in KCs promoted the polarization of KCs towards the M1 phenotype and increased the expression of TNF-*α*. The increase in KC-derived TNF-*α* triggered the apoptosis of HSCs and the activation of TNF-R1/caspase 8 in vitro, which was consistent with the in vivo results.

**Conclusion:**

ER stress in KCs promotes the polarization of these cells towards the M1 phenotype and increases the expression of TNF-*α*. Then, the increase in KC-derived TNF-*α* triggers the apoptosis of HSCs through TNF-R1/caspase 8.

## 1. Introduction

Hepatic fibrosis is a self-healing process caused by multiple chronic liver injuries. Hepatic fibrosis is characterized by the deposition of large amounts of extracellular matrix, which leads to the abnormal proliferation of connective tissue in the liver [1]. In this pathological progression, liver fibrosis is the only reversible process. Hence, it is particularly important to explore methods of reversing liver fibrosis to prevent hepatocellular carcinoma, which is caused by liver cirrhosis [2]. Many previous studies have shown that active HSCs play an important role in the progression of hepatic fibrosis. Panebianco et al. [3] reviewed the role of retinoic acid signaling in modulating the fibrogenic potential of HSCs and proposed that it acts in synergy with PPAR-*γ* in the reversal of liver fibrosis. Liu et al. [4] reported that osthole improves TAA-induced liver injury, fibrogenesis, and inflammation in rats by suppressing HSC activation. Therefore, inducing the apoptosis of active HSCs is an important strategy for blocking the development of hepatic fibrosis.

Endoplasmic reticulum stress (ER stress) is an imbalance of ER homeostasis caused by the overactivation of the unfolded protein response. Previous studies have shown that ER stress plays an important regulatory role in inducing the apoptosis of active HSCs. Wang et al. [5] postulated that etoposide induces apoptosis in activated human HSCs via ER stress. Li et al. [6] reported that ER stress-mediated autophagy enhances the caffeine-induced apoptosis of HSCs. However, some studies have shown that ER stress does not induce the apoptosis of active HSCs [7]. Thus, clarifying the mechanism by which ER stress induces the apoptosis of active HSCs requires further study.

KCs, which are macrophages located in the liver, are a kind of nonparenchymal liver cell. Due to their high plasticity, KCs can play different roles in different microenvironments [8–10]. The ER stress in KCs is associated with the secretion of various inflammatory factors, especially TNF-*α*. A previous study [11] showed that the secretion of TNF-*α* by bone marrow-derived macrophages is increased due to ER stress. Xu et al. [12] also reported that TUDCA can reduce the secretion of TNF-*α* from KCs by inhibiting ER stress. Remarkably, TNF-*α* is widely believed to be involved in the regulation of apoptosis in multiple cell types [13–15], but whether ER stress-induced TNF-*α* secretion by KCs has an effect on the apoptosis of active HSCs has not been reported.

In this study, we investigated the effect of ER stress-induced TNF-*α* production by KCs on the apoptosis of active HSCs, as well as the possible underlying mechanism, and aimed to identify new treatments to reverse the progression of hepatic fibrosis.

## 2. Materials and Methods

### 2.1. Materials

Dulbecco's modified Eagle's medium (DMEM) and enzyme-linked immunosorbent assay (ELISA) kits for TNF-*α* were obtained from Abcam Trading Company, Ltd. (Shanghai, China). A terminal deoxynucleotidyl transferase-mediated dUTP-biotin nick-end labeling (TUNEL) kit was purchased from Roche Diagnostics (Roche, Shanghai, China). The relevant antibody information is shown in Table 1. All the other reagents used in this study are commercially available and were of analytical grade.

### 2.2. Animals and Protocols

Male SD rats (8-10 weeks old, each weighing 267 ± 33.2 g) were provided by the Experimental Animal Center of Chongqing Medical University (Chongqing, China). Humane care was provided to all the animals, according to the National Institutes of Health. The protocols used in this research were evaluated and approved by the Animal Use and Ethics Committee of the 2nd Affiliated Hospital of Chongqing Medical University (2015–2018). The rats were randomly divided into 4 groups as follows:

(1) Control group (n = 15). The rats were treated with normal saline (2 mg/kg) by intraperitoneal injection twice a week for 8 weeks. The rats were allowed to drink, eat, exercise, and rest freely after administration.

(2) Model group (*n* = 15). The rats were treated with 10% CCl_4_ solution (made using peanut oil) by intraperitoneal injection twice a week for 8 weeks. The rats were allowed to drink, eat, exercise, and rest freely after administration.

(3) ER stress group (*n* = 15). The rats were treated with 10% CCl_4_ solution and tunicamycin (1 mg/kg) [16] by intraperitoneal injection twice a week for 8 weeks. The rats were allowed to drink, eat, exercise, and rest freely after administration.

(4) Depletion group (*n* = 15). The rats were treated with 10% CCl_4_ solution and tunicamycin (1 mg/kg) by intraperitoneal injection twice a week for 8 weeks. Liposomal clodronate (50 mg/kg) was intraperitoneally injected following treatment with tunicamycin [17]. The rats were allowed to drink, eat, exercise, and rest freely after administration.

The rats in each group were assigned to the following subgroups: 3 rats were used to make liver tissue sections, 3 rats were used to isolate primary Kupffer cells, 3 rats were used to isolate primary hepatic stellate cells, and 6 rats were used to extract tissue proteins and RNA.

### 2.3. Establishment of the Cell Model

#### 2.3.1. Isolation and Culture of KCs

Primary KCs were isolated from male SD rats at 8-10 weeks of age by a three-step approach: digestion by collagenase IV, density gradient centrifugation, and selective adherence [17]. The KCs were cultured in DMEM supplemented with 10% FBS, 100 U/ml penicillin G, and 100 U/ml streptomycin at 37°C in the presence of 5% CO_2_.

#### 2.3.2. Isolation of Hepatic Stellate Cells

Primary HSCs were isolated from male SD rats at 8-10 weeks of age by a combination of pronase collagenase digestion, density gradient centrifugation, and centrifugal elutriation as previously described [18]. The cells were cultured in DMEM supplemented with 10% FBS, 100 U/ml penicillin G, and 100 U/ml streptomycin at 37°C in the presence of 5% CO_2_.

#### 2.3.3. TNF-*α* and TGF-*β* Treatment of Hepatic Stellate Cells

To determine the effect of TNF-*α* on HSC activation, HSCs were isolated as described above and treated with TNF-*α* at different concentrations (10 ng/ml, 20 ng/ml, and 40 ng/ml) for 12 h. After 12 h of treatment, HSC and protein samples were collected for the subsequent experiments.

To observe the effect of TNF-*α* on the apoptosis of activated hepatic stellate cells, HSCs were isolated as described above and treated with TGF-*β* (5 ng/ml) for 24 h. Then, the HSCs were treated with TNF-*α* at different concentrations (10 ng/ml, 20 ng/ml, and 40 ng/ml) for 12 h. After 12 h of treatment, HSC and protein samples were collected for the subsequent experiments.

#### 2.3.4. Coculture of KCs and HSCs

KCs and hepatic stellate cells were isolated according to the methods described above. Then, the hepatic stellate cells were cocultured with the KCs from each group (at 10 : 1) in DMEM supplemented with 10% FBS, 100 U/ml penicillin G, and 100 U/ml streptomycin at 37°C in the presence of 5% CO_2_. The two cell types are separated by a transwell chamber to ensure that there was no direct contact between the cells and only an exchange of cell supernatants. After 24 h, the primary hepatic stellate cells and cellular proteins were collected for the subsequent experiments.

### 2.4. ELISA

TNF-*α* in the KC supernatants was measured by ELISA. Antigen serum was added to an ELISA plate (100 *μ*l/well) and incubated at room temperature overnight. The serum or supernatant samples were then added to the ELISA plate (100 *μ*l/well) and incubated at 37°C for 1 h. Then, IgG antibody was added, and the plate was again incubated for 1 h. Subsequently, a working solution was added to the ELISA plate, and the plate was incubated for 30 min at 37°C. Finally, a color reagent was added to the ELISA plate for detection. The absorbance index of each plate was detected by an ELISA plate reader.

### 2.5. Immunofluorescence Staining

KCs and hepatic stellate cells were used to generate cell slides. The samples were fixed with 4% buffered formaldehyde, permeabilized with 0.2% Triton X-100, and blocked with 5% BSA at room temperature. The following incubations were performed in the dark. The KCs were incubated at 4°C overnight with primary antibodies against CD68 (1 : 200), CD16 (1 : 200), and desmin (1 : 200). The KCs were then incubated with a secondary antibody (1 : 5000) at room temperature for 1 h, followed by a final incubation with DAPI at room temperature.

The liver tissues were made into frozen sections. The sections were thawed at room temperature, washed with PBS, fixed with 4% buffered formaldehyde, permeabilized with 0.2% Triton X-100, and blocked with 5% BSA at room temperature. The following incubations were performed in the dark. The sections were incubated at 4°C overnight with primary antibodies against desmin (1 : 200). The sections were then incubated with a secondary antibody (1 : 2000) at room temperature for 1 h, followed by a final incubation with DAPI at room temperature.

### 2.6. Western Blot Analysis

Western blot analysis was performed as follows: the protein samples were electrophoresed and transferred to membranes. Then, the membranes were blocked. Next, the membranes were incubated at 4°C overnight with primary antibodies and incubated at room temperature with secondary antibodies for 1 h. The relative expression of the protein of interest was normalized to the expression of GAPDH.

### 2.7. Examination of Liver Function

Blood samples were collected from each group after the operation was performed. Serum liver function markers, including alanine aminotransferase (AST) and aspartate transaminase (ALT), were detected with an automatic biochemical analyzer (Beckman CX7, Beckman Coulter, CA, USA) to evaluate liver function.

### 2.8. Sirius Red Staining

Paraffin sections were dewaxed with xylene and eluted using different concentrations of ethanol. Then, the paraffin sections were stained with Sirius red solution and hematoxylin and blocked with neutral balsam.

### 2.9. Immunohistochemical Staining

Paraffin sections were dewaxed with xylene and eluted using different concentrations of ethanol. The sections then underwent antigen repair with a sodium citrate solution, blocking with goat serum and incubation with primary antibodies against *α*-SMA (1 : 500), CD16 (1 : 500), and desmin (1 : 640) at 4°C overnight. Then, the paraffin sections were incubated with a secondary antibody at 37°C for 30 min and stained with a DAB solution. Hematoxylin was then added to the sections at room temperature for 30 s; then, the sections were dewaxed with xylene and again eluted in gradients of ethyl alcohol.

### 2.10. TUNEL Immunostaining

TUNEL immunostaining was performed according to the manufacturer's instructions. The staining of the cell nuclei in each group was observed by fluorescence microscopy.

### 2.11. Flow Cytometry

The collected cells were placed in staining buffer to form a cell suspension. Then, the cell suspension was centrifuged at 300 × g for 5 min. A fluorescently labeled antibody (CD16 at 1 : 100 or Arg1 at 1 : 60) was added to the cell suspension at the concentration recommended in the manufacturer's instructions and incubated for 30 min in the dark. Then, the cell suspension was again centrifuged at 300 × g for 5 min. The cells were resuspended in cell staining buffer and then detected and analyzed by flow cytometry.

### 2.12. Statistical Analysis

All the results were analyzed using SPSS 18.0 software (SPSS Inc., Chicago, USA). Normally distributed data are shown as the mean ± SD. Differences between groups were detected using a *t* test. The Shapiro-Wilk test was used to test normality. Data with a sig value >0.05 were regarded as conforming to a normal distribution. Nonnormally distributed data are shown as the median; differences were detected using the rank-sum test. Differences with *P* values < 0.05 were regarded as statistically significant.

## 3. Results

### 3.1. ER Stress in KCs Decreased the Levels of Liver Function Markers and the Degree of Liver Fibrosis in a Rat Model

**Figure 1:**
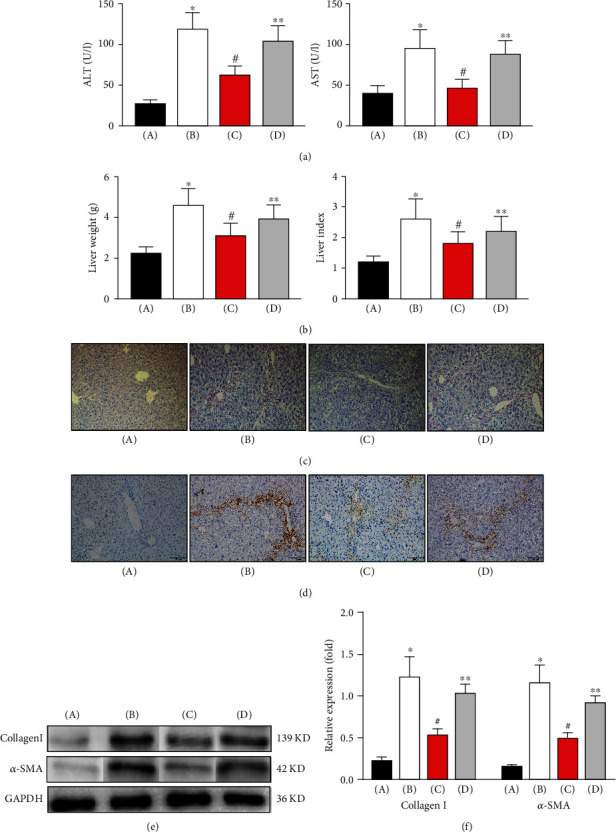
ER stress in KCs decreased the levels of liver function markers and the degree of liver fibrosis in a rat model. (a) The serum levels of AST and ALT in each group. (b) The liver weight and liver indexes in each group. (c) The liver fibrosis in each group was detected by Sirius Red staining. (d) The *α*-SMA expression in each group was detected by immunohistochemical staining. (e) The collagen I and *α*-SMA expression in each group was detected by Western blot. (f) Quantitative analysis of Western blots. ^∗^Model group vs. control group. ^#^ER stress group vs. model group. ^∗∗^ER stress group vs. depletion group, *P* < 0.05.

Liver and blood samples were collected from the different groups to investigate the levels of liver function markers and the degree of liver fibrosis. First, the serum levels of liver function markers and liver weight were determined. As shown in Figure 1(a), compared with those in the control group, the levels of ALT and AST in the model group were markedly increased (*P* < 0.05). In response to tunicamycin treatment, compared with those in the model group, the levels of ALT and AST in the ER stress group were markedly decreased (*P* < 0.05). After KC depletion, the levels of ALT and AST in the depletion group were markedly higher than those in the ER stress group (*P* < 0.05). The liver weight and liver index in each group, as shown in Figure 1(b), displayed similar tendencies. Then, the degree of liver fibrosis was detected by Sirius Red staining. As shown in Figure 1(c), compared with that in the control group, the positive region in the model group was markedly increased (*P* < 0.05). In response to tunicamycin treatment, compared with that in the model group, the positive region in the ER stress group was markedly decreased (*P* < 0.05). After KC depletion, compared with that in the ER stress group, the positive region in the depletion group was markedly increased (*P* < 0.05). We also detected the expression of fibrosis-associated proteins, including collagen I and *α*-SMA, by immunohistochemical staining and Western blotting. As shown in Figure 1(d), compared with that in the control group, the *α*-SMA-positive region in the model group was markedly increased (*P* < 0.05). In response to tunicamycin treatment, compared with that in the model group, the *α*-SMA- positive region in the ER stress group was markedly decreased (*P* < 0.05). After the KCs were depleted, compared with that in the ER stress group, the *α*-SMA-positive region in the depletion group was markedly increased (*P* < 0.05). As shown in Figures 1(e) and 1(f), the levels of collagen I and *α*-SMA displayed similar tendencies. According to these data, we concluded that ER stress in KCs decreases the levels of liver function markers and the degree of liver fibrosis.

### 3.2. ER Stress in KCs Increased the Number of Cells with the M1 Phenotype and the Secretion of TNF-*α* and Reduced the Number of Active HSCs in a Rat Model

**Figure 2:**
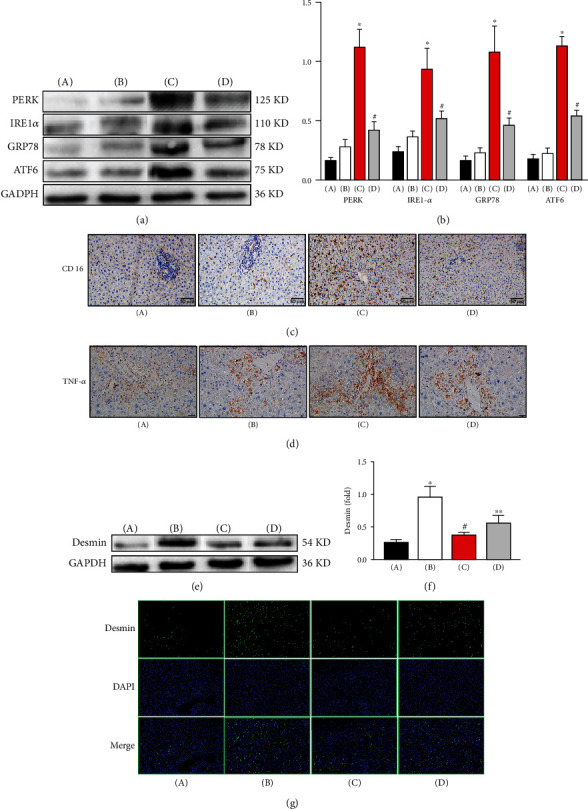
ER stress in KCs increased the number of KCs with an M1 phenotype and the secretion of TNF-*α* and reduced the number of active HSCs in a rat model. (a) The levels of PERK, IRE1*α*, GRP78, and ATF6 were detected by Western blot. (b) Quantitative analysis of Western blots. (c) The M1 phenotype of KCs was detected by immunohistochemical staining. (d) The serum level of TNF-*α* was detected by immunohistochemical staining. (A) Control group, (B) model group, (C) ER stress group, and (D) depletion group. ^#^ER stress group vs. model group, ^∗∗^ER stress group vs. depletion group, *P* < 0.05. (e) The level of desmin was detected by Western blot. (f) Quantitative analysis of Western blots. (g) Active HSCs were detected by immunohistochemical staining. (A) Control group, (B) model group, (C) ER stress group, and (D) depletion group. ^∗^Model group vs. control group. ^#^ER stress group vs. model group. ^∗∗^ER stress group vs. depletion group, *P* < 0.05.

Next, we investigated whether ER stress in KCs exerts a protective effect by affecting active HSCs. First, the levels of ER stress-related proteins, including PERK, IRE1*α*, GRP78, and ATF6, were detected by Western blot. As shown in Figures 2(a) and 2(b), compared with those in the control group, the levels of the above proteins in the model group were not significantly different (*P* > 0.05). In response to tunicamycin treatment, compared with those in the model group, the levels of the above proteins in the ER stress group were markedly increased (*P* < 0.05). After KC depletion, compared with those in the ER stress group, the levels of the above proteins in the depletion group were markedly decreased (*P* < 0.05). Then, the number of KCs with the M1 phenotype and the level of TNF-*α* in the liver were detected by immunohistochemical staining. As shown in Figures 2(c) and 2(d), compared with those in the control group, the number of CD16-positive KCs and the level of TNF-*α* in the model group were not significantly different (*P* > 0.05). In response to tunicamycin treatment, compared with those in the model group, the number of CD16-positive KCs and the level of TNF-*α* were markedly increased (*P* < 0.05). After KC depletion, compared with those in the ER stress group, the number of CD16-positive KCs and the level of TNF-*α* were markedly decreased (*P* < 0.05). The level of desmin was detected by Western blot. As shown in Figures 2(e) and 2(f), compared with that in the control group, the level of desmin in the model group was markedly increased (*P* < 0.05). In response to tunicamycin treatment, compared with that in the model group, the level of desmin was markedly decreased (*P* < 0.05). After KC depletion, compared with that in the ER stress group, the level of desmin was markedly increased (*P* < 0.05). Furthermore, the number of active HSCs was also assessed by immunohistochemical staining. As shown in Figure 2(g), compared with that in the control group, the number of desmin-positive cells in the model group was markedly increased (*P* < 0.05). In response to tunicamycin treatment, compared with that in the model group, the number of desmin-positive cells was markedly decreased (*P* < 0.05). After KC depletion, compared with that in the ER stress group, the number of desmin-positive cells in the depletion group was markedly increased (*P* < 0.05). Our data show that, in a rat model, ER stress in KCs exerts its protective effect by increasing the number of cells with the M1 phenotype and the secretion of TNF-*α* and then reducing the number of active HSCs.

### 3.3. The Number of Active HSCs Decreased Mainly due to TNF-*α*-Induced Apoptosis

**Figure 3:**
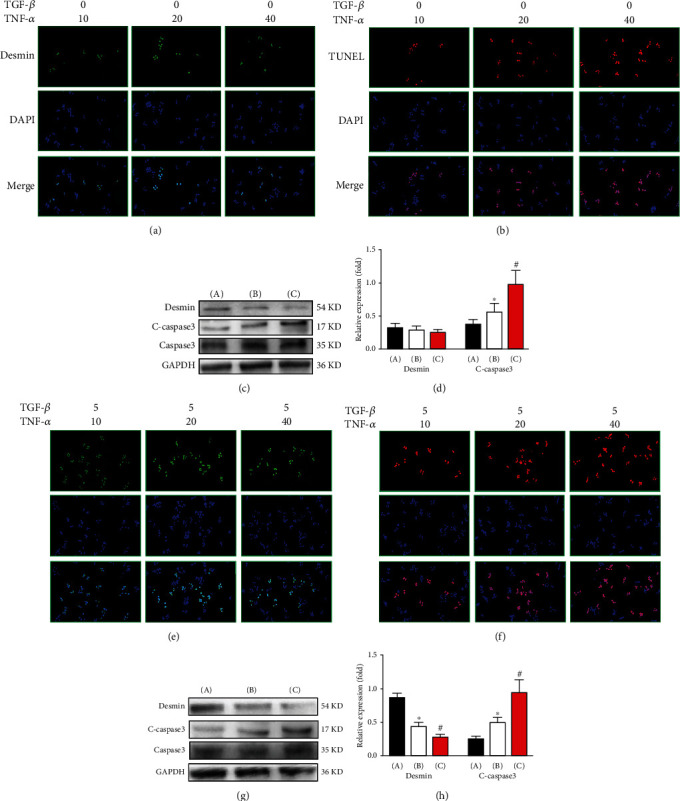
The number of active HSCs decreased mainly due to apoptosis induced by ER stress in KCs. (a) The activation of stationary HSCs in each group was detected by immunofluorescence staining. (b) The apoptosis of stationary HSCs in each group was detected by TUNEL staining. (c) The levels of desmin and C-caspase 3 in each group were detected by Western blot. (d) Quantitative analysis of Western blots. (A) 10 ng/ml TNF-*α* group, (B) 20 ng/ml TNF-*α* group, and (C) 40 ng/ml TNF-*α* group. (e) The activation of HSCs in each group was detected by immunofluorescence staining. (f) The apoptosis of activated HSCs in each group was detected by TUNEL staining. (g) The levels of desmin and C-caspase 3 in each group were detected by Western blot. (h) Quantitative analysis of Western blots. (A) 10 ng/ml TNF-*α* group, (B) 20 ng/ml TNF-*α* group, and (C) 40 ng/ml TNF-*α* group. ^∗^20 ng/ml TNF-*α* group vs. 10 ng/ml TNF-*α* group, ^#^40 ng/ml TNF-*α* group vs. 20 ng/ml TNF-*α* group, *P* > 0.05.

To determine the effect of TNF-*α* on stationary HSC activation, primary HSCs were treated with TNF-*α* at different concentrations (10 ng/ml, 20 ng/ml, and 40 ng/ml). After 12 h of treatment, desmin-specific immunofluorescence staining was used to detect the degree of stationary hepatic stellate cell activation in each group. As shown in Figure 3(a), the number of desmin-positive HSCs in each group was not significantly different (*P* > 0.05). Then, TUNEL staining was used to detect the degree of apoptosis of stationary hepatic stellate cells in each group. As shown in Figure 3(b), compared with that in the 10 ng/ml TNF-*α* group, the number of TUNEL-positive HSCs in the 20 ng/ml TNF-*α* group was markedly increased (*P* < 0.05). Compared with that in the 20 ng/ml TNF-*α* group, the number of TUNEL-positive HSCs in the 40 ng/ml TNF-*α* group was markedly increased (*P* < 0.05). In addition, the expression of desmin and C-caspase 3 in the hepatic stellate cells of each group was detected by Western blot. As shown in Figures 3(c) and 3(d), compared with that in the 10 ng/ml TNF-*α* group, the level of desmin in each group was not significantly different (*P* > 0.05). Compared with that in the 10 ng/ml TNF-*α* group, the level of C-caspase 3 in the 20 ng/ml TNF-*α* group was markedly increased (*P* < 0.05). Compared with that in the 20 ng/ml TNF-*α* group, the level of C-caspase 3 in the 40 ng/ml TNF-*α* group was markedly increased (*P* < 0.05). These data indicate that in our model, TNF-*α* has no obvious effect on activating stationary hepatic stellate cells but has an obvious effect on promoting apoptosis.

Then, we further observed the effect of TNF-*α* on the apoptosis of activated hepatic stellate cells. Stationary HSCs were treated with TGF-*β* (5 ng/ml) for 24 h. Then, activated HSCs were treated with TNF-*α* at different concentrations (10 ng/ml, 20 ng/ml, and 40 ng/ml). After 12 h of treatment, desmin-specific cell immunofluorescence staining was used to detect the degree of hepatic stellate cell activation in each group. As shown in Figure 3(e), compared with that in the 10 ng/ml TNF-*α* group, the number of desmin-positive HSCs in the 20 ng/ml TNF-*α* group was markedly decreased (*P* < 0.05). Compared with that in the 20 ng/ml TNF-*α* group, the number of desmin-positive HSCs in the 40 ng/ml TNF-*α* group was markedly decreased (*P* < 0.05). Then, TUNEL staining was used to detect the degree of apoptosis of the hepatic stellate cells in each group. Compared with that in the 10 ng/ml TNF-*α* group, the number of TUNEL-positive HSCs in the 20 ng/ml TNF-*α* group was markedly increased (*P* < 0.05). Compared with that in the 20 ng/ml TNF-*α* group, the number of TUNEL-positive HSCs in the 40 ng/ml TNF-*α* group was markedly increased (*P* < 0.05). In addition, the expression of desmin and C-caspase 3 in the hepatic stellate cells of each group was detected by Western blot. As shown in Figures 3(f) and 3(g), compared with that in the 10 ng/ml TNF-*α* group, the level of desmin in the 20 ng/ml TNF-*α* group was markedly decreased (*P* < 0.05). Compared with that in the 20 ng/ml TNF-*α* group, the level of desmin in the 40 ng/ml TNF-*α* group was markedly decreased (*P* < 0.05). Compared with that in the 10 ng/ml TNF-*α* group, the level of C-caspase 3 in the 20 ng/ml TNF-*α* group was markedly increased (*P* < 0.05). Compared with that in the 20 ng/ml TNF-*α* group, the level of C-caspase 3 in the 40 ng/ml TNF-*α* group was markedly increased (*P* < 0.05). These data indicate that in our model, TNF-*α* has an obvious apoptosis-promoting effect on activated HSCs.

Hence, we believed that the number of active HSCs decreased mainly due to TNF-*α*-induced apoptosis.

### 3.4. ER Stress May Promote the Polarization of KCs toward the M1 Phenotype via the IRE1*α*/TRAF2/NF-*κ*B Signaling Pathway

**Figure 4:**
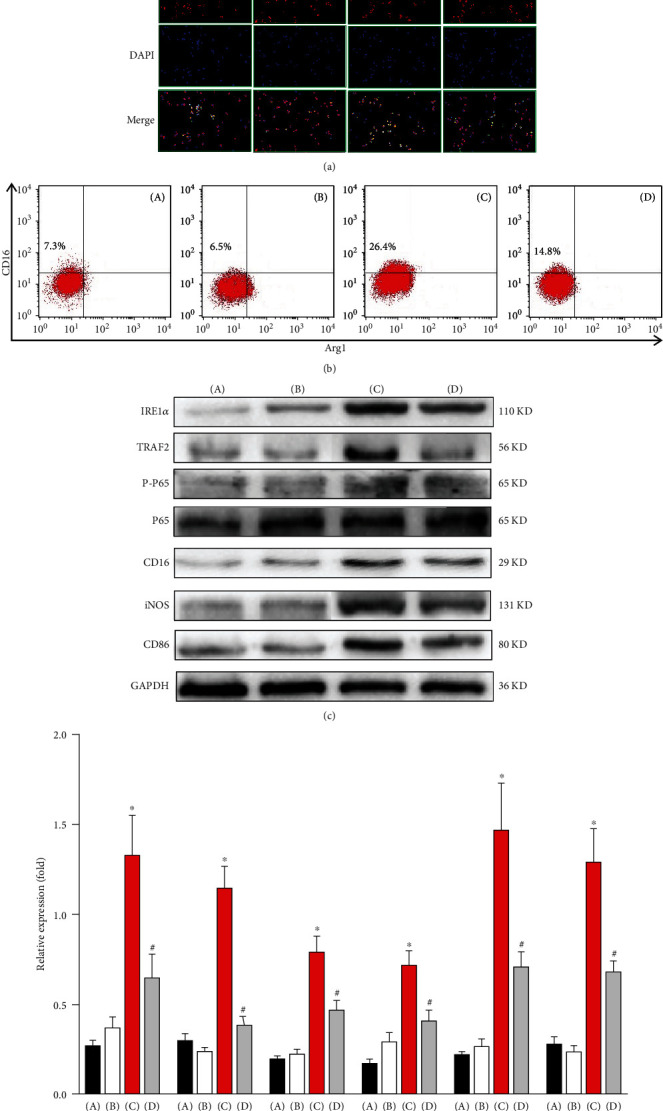
ER stress promotes the polarization of KCs toward the M1 phenotype via the IRE1/TRAF2/NF-*κ*B signaling pathway. (a) The M1 phenotype of KCs was detected by dual immunofluorescence staining. CD16 (green), CD68 (red), and DAPI (blue). (b) The proportion of KCs with an M1 phenotype was detected by CD16/Arg1 staining and assessed by flow cytometry. (c) The levels of IRE1*α*, TRAF2, P-P65, CD16, iNOS, and CD86 were detected by Western blot. (d) Quantitative analysis of Western blots. (A) Control group, (B) model group, (C) ER stress group, and (D) depletion group. ^∗^ER stress group vs. model group. ^#^ER stress group vs. depletion group, *P* < 0.05.

To further elucidate the effect of ER stress on KC polarization, we isolated and cultured KCs from each group and analyzed their polarization status. First, dual immunofluorescence staining was utilized to detect the proportion of KCs with an M1 phenotype. As shown in Figure 4(a), compared with that in the control group, the number of CD16/CD68 double-positive KCs in the model group was not significantly different (*P* > 0.05). In response to tunicamycin treatment, compared with that in the model group, the number of CD16/CD68 double-positive KCs was markedly increased (*P* < 0.05). After KC depletion, compared with that in the ER stress group, the number of CD16/CD68 double-positive KCs was markedly decreased (*P* < 0.05). We also examined the proportion of KCs with an M1 phenotype by flow cytometry. As shown in Figure 4(b), compared with that in the control group, the number of CD16-positive/Arg1-negative KCs in the model group was not significantly different (*P* > 0.05). In response to tunicamycin treatment, compared with that in the model group, the number of CD16-positive/Arg1-negative KCs was markedly increased (*P* < 0.05). After KC depletion, compared with that in the ER stress group, the number of CD16-positive/Arg1-negative KCs was markedly decreased (*P* < 0.05). These data indicate that ER stress promotes the polarization of KCs toward the M1 phenotype.

Then, we explored the possible mechanism by which ER stress affects KC polarization. The levels of IRE1*α*, TRAF2, P-P65, CD16, iNOS, and CD86 were detected by Western blot. As shown in Figures 4(c) and 4(d), compared with those in the control group, the levels of IRE1*α*, TRAF2, P-P65, CD16, iNOS, and CD86 in the model group were not significantly different (*P* > 0.05). In response to tunicamycin treatment, compared with those in the model group, the levels of IRE1*α*, TRAF2, P-P65, CD16, iNOS, and CD86 were markedly increased (*P* < 0.05). After the depletion of KCs, compared with those in the ER stress group, the levels of IRE1*α*, TRAF2, P-P65, CD16, iNOS, and CD86 were markedly decreased (*P* < 0.05). In conclusion, our study preliminarily suggests that ER stress promotes the polarization of KCs toward the M1 phenotype via the IRE1*α*/TRAF2/NF-*κ*B signaling pathway.

### 3.5. KC-Derived TNF-*α* May Trigger the Apoptosis of Activated HSCs by Promoting the Activation of TNF-R1/Caspase 8

**Figure 5:**
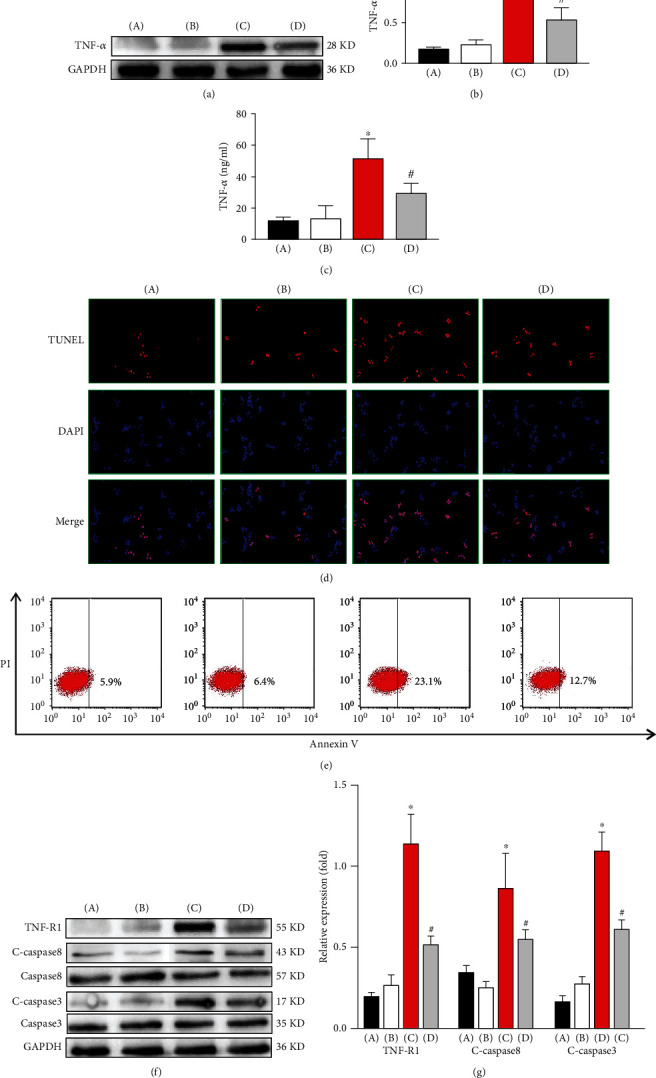
Kupffer cell-derived TNF-*α* triggers the apoptosis of HSCs by promoting the activation of TNF-R1/caspase 8. (a) The level of TNF-*α* in each group of KCs by Western blot. (b) Quantitative analysis of Western blots. (c) The levels of TNF-*α* in the cell supernatants were analyzed by ELISA. (d) The apoptosis of the activated HSCs in each group was detected by TUNEL staining. (e) Flow cytometry was also utilized to examine the apoptosis of active HSCs. (f) The expression of TNF-R1, C-caspase 8, and C-caspase 3 in each group. (g) Quantitative analysis of Western blots. (A) Control group, (B) model group, (C) ER stress group, and (D) depletion group. ^∗^ER stress group vs. model group. ^#^Depletion group vs. ER stress group, *P* < 0.05.

To further investigate the role of ER stress in KCs in TNF-*α* secretion and activated HSC apoptosis, we isolated and cultured KCs from each group and cocultured them with active HSCs (treated with TGF-*β* at concentrations of 5 ng/ml). First, we examined the level of TNF-*α* in the KCs from each group by Western blot. As shown in Figures 5(a) and 5(b), compared with that in the control group, the level of TNF-*α* in the model group was not significantly different (*P* > 0.05). In response to tunicamycin treatment, compared with that in the model group, the level of TNF-*α* was markedly increased (*P* < 0.05). After the depletion of KCs, compared with that in the ER stress group, the level of TNF-*α* was markedly decreased (*P* < 0.05). Furthermore, the level of TNF-*α* in the cell supernatant was tested by ELISA. As shown in Figure 5(c), the results showed similar trends.

In the KC cocultures, the apoptosis of the activated HSCs in each group was detected by TUNEL staining. As shown in Figure 5(d), compared with that in the control group, the number of apoptotic HSCs in the model group was not significantly different (*P* > 0.05). In response to tunicamycin treatment, compared with that in the model group, the number of apoptotic HSCs was markedly increased (*P* < 0.05). After the depletion of KCs, compared with that in the ER stress group, the number of apoptotic HSCs was markedly decreased (*P* < 0.05). Flow cytometry was also utilized to examine the apoptosis of active HSCs. As shown in Figure 5(e), the results showed similar trends.

Then, we explored the underlying mechanism by which KC-derived TNF-*α* triggers the apoptosis of activated HSCs. The levels of TNF-R1, C-caspase 8, and C-caspase 3 were detected by Western blot. As shown in Figures 5(f) and 5(g), compared with those in the control group, the levels of the abovementioned proteins in the model group were not significantly different (*P* > 0.05). In response to tunicamycin treatment, compared with those in the model group, the levels of the abovementioned proteins were markedly increased (*P* < 0.05). After the depletion of KCs, compared with those in the ER stress group, the levels of the abovementioned proteins were markedly decreased (*P* < 0.05).

Thus, we conclude that ER stress induced by tunicamycin may promote the polarization of KCs toward the M1 phenotype and the secretion of TNF-*α*. KC-derived TNF-*α* then triggers the apoptosis of activated HSCs by promoting the activation of TNF-R1/caspase 8.

## 4. Discussion

Liver cirrhosis is the terminal stage of progressive liver fibrosis and is estimated to affect 1% to 2% of the global population, resulting in over 1 million deaths worldwide each year [16, 19]. Remarkably, in the pathogenesis of liver fibrosis, activated HSCs are the major cellular source of matrix protein-secreting myofibroblasts and the major drivers of liver fibrogenesis [20, 21]. Currently, there are two main options for treating liver fibrosis that target HSCs. One treatment option is to inhibit the activation of HSCs. The other treatment option is to promote the apoptosis of HSCs. In our study, with the CCl_4_-induced apoptosis of active HSCs, the serum levels of AFT and ALT and the degree of liver fibrosis became markedly decreased. Furthermore, the serum level of TNF-*α* also declined. These results suggest that promoting the apoptosis of active HSCs can reduce the severity of hepatic fibrosis, which is consistent with the results of a number of previous studies [22].

TNF-*α* is an important cytokine that is widely believed to be involved in the regulation of apoptosis. Grabinger et al. [23] found that cIAP1 is required for the regulation of TNF-induced intestinal epithelial cell death and survival. An experiment with heart muscle cells suggested that TNF-*α*-induced AIF upregulation contributes to apoptosis in rat primary cardiomyocytes [24]. Therefore, we speculated that promoting TNF-*α* secretion in the liver might be an important way to induce apoptosis of activated HSCs. In our research, we found that with increased TNF-*α* expression in liver tissue, the number of activated HSCs in liver tissue significantly decreased. This result may be due to the increased apoptosis of activated HSCs or to the decreased activation of inactive HSCs. The most accurate method for determining the reason for the decrease in the number of activated stellate cells in liver tissue is to detect the absolute number of apoptotic activated stellate cells in liver tissue. However, we have not found an appropriate antibody for labeling both HSCs and apoptotic cells (common hepatic stellate cell markers, such as desmin and *α*-SMA, are expressed in activated HSCs, while apoptotic HSCs do not express these markers). Therefore, in this experiment, we failed to directly detect HSC apoptosis in fibrotic tissue, which is a deficiency in our experiment.

To compensate for this deficiency, we isolated primary HSCs from rats and further verified the effect of TNF-*α* on the apoptosis of hepatic stellate cells. Our experimental results show that in our model, TNF-*α* has an obvious apoptosis-promoting effect on stationary HSCs. Moreover, TNF-*α* has an obvious apoptosis-promoting effect on activated HSCs. This effect is supported by previous studies [25–27]. TNF-*α* can directly promote the apoptosis of or inhibit the proliferation of HSCs, especially in a chronic hepatic fibrosis model. However, it has also been reported that TNF-*α* can promote the proliferation of HSCs through the NF-*κ*B pathway, especially in an acute hepatic injury model [4, 28]. We think that the possible causes of this difference are mainly explained by the following differences in the models of liver fibrosis: first, there are differences in the amount of TNF-*α* secretion, and in our model, 40 ng/ml TNF-*α* promotes apoptosis; second, TNF-*α* causes the activation of different downstream signaling pathways, and in our model, TNF-*α* mainly stimulates the activation of the TNF-R1/caspase 8 pathway, not the NF-*κ*B signaling pathway. In addition, some studies have suggested that TNF-*α* can cause hepatocyte apoptosis and hepatic injury, especially in the acute inflammatory phase, and then, hepatocyte apoptosis and hepatic injury trigger the activation and proliferation of HSCs [29, 30]. In summary, we established a model of chronic liver fibrosis, which is different from the model of acute liver injury. In our model, TNF-*α* may mainly directly promote apoptosis through the TNF-R1/caspase 8 pathway.

KCs, which are macrophages located in the liver, are a kind of nonparenchymal liver cell. Due to their high plasticity, KCs can play different roles in different microenvironments. Previous studies [31, 32] have found that M2 macrophages can secrete a large amount of TGF-*β*, thus activating hepatic stellate cells and promoting the progression of parenchymal organ fibrosis, including liver fibrosis. Blocking the function of KCs can reduce the degree of liver fibrosis, but it mainly blocks the function of M2 KCs. Therefore, the induction of KC apoptosis is considered to be an important method to reduce liver fibrosis. However, previous studies have found that KCs have obvious antiapoptotic effects in the microenvironment of liver fibrosis [33]. In addition, it is worth noting that M1 macrophages are thought to reduce the degree of liver fibrosis [34]. Therefore, we speculate that compared with blocking the function of KCs or inducing the apoptosis of KCs, promoting the polarization of KCs from the M2 phenotype to the M1 phenotype may be more effective in reducing the degree of liver fibrosis.

ER stress is an abnormal state of function and stress caused by the accumulation of unfolded or misfolded proteins in the endoplasmic reticulum. Remarkably, previous studies [11, 12] found that because they are important secretors of TNF-*α* in the liver [35], inhibiting ER stress in KCs can reduce the number of M1 KCs and the amount of TNF-*α* secretion in the liver. In this study, we found that in response to treatment with tunicamycin, the number of M1-like KCs and the level of TNF-*α* were increased. ER stress in KCs markedly decreased the degree of hepatic fibrosis and the serum levels of ALT and AST. Furthermore, the number of active HSCs in the liver was also markedly decreased. All the above results were reversed upon the depletion of KCs. Our data show that ER stress in KCs can reduce the number of active HSCs in the liver by increasing the secretion of TNF-*α*. Furthermore, we investigated the possible mechanism by which KC-derived TNF-*α* triggers the apoptosis of active HSCs. We found that the number of active HSCs decreased mainly due to apoptosis induced by ER stress in KCs. Furthermore, the expression of TNF-R1, cleaved caspase 8, and cleaved caspase 3 in HSCs was markedly increased. These data indicate that KC-derived TNF-*α* triggers the apoptosis of HSCs by promoting the activation of TNF-R1/caspase 8.

In conclusion, we have shown that ER stress induced by tunicamycin promotes the M1 polarization of KCs and the secretion of TNF-*α*. Kupffer cell-derived TNF-*α* then triggers the apoptosis of HSCs by promoting the activation of TNF-R1/caspase 8.

## Figures and Tables

**Table 1:** The relevant antibody information.

Name	Catalog numbers	Company
*α*-SMA	ab32575	Abcam
Collagen-I	ab34710	Abcam
PERK	ab229912	Abcam
GRP78	ab21685	Abcam
ATF6	ab203119	Abcam
IRE1*α*	ab37073	Abcam
CD16	ab203883	Abcam
TNF-*α*	ab66579	Abcam
Desmin	ab32362	Abcam
C-caspase 3	ab49822	Abcam
Caspase 3	ab44976	Abcam
CD68	ab31630	Abcam
TRAF2	ab126758	Abcam
P65	ab32536	Abcam
P-P65	ab86299	Abcam
TNF-R1	#13377	CST
C-caspase 8	ab227430	Abcam
Caspase 8	ab108333	Abcam
GAPDH	#5174	CST
Arg1	ab233548	Abcam

## Data Availability

The datasets used and/or analyzed during the current study are available from the corresponding author upon reasonable request.

## Abbreviations

ER: Endoplasmic reticulum
KCs: Kupffer cells
HSC: Hepatic stellate cell
ELISA: Enzyme-linked immunosorbent assay
TUNEL: Terminal deoxynucleotidyl transferase-mediated dUTP-biotin nick-end labeling
DMEM: Dulbecco's modified Eagle's medium
FBS: Fetal bovine serum
AST: Alanine aminotransferase
ALT: Aspartate transaminase
TNF-*α*: Tumor necrosis factor-*α*
PERK: Protein kinase R- (PKR-) like endoplasmic reticulum kinase
IRE1*α*: Inositol-requiring enzyme-1
GRP78: Glucose-regulated protein 78
ATF6: Activating transcription factor 6
TGF-*β*: Transcription growth factor
TRAF2: TNF receptor-associated factor 2
TNF-R1: TNF receptor 1.

## References

[1] Bitto N., Liguori E., La Mura V. (2018). Coagulation, microenvironment and liver fibrosis. *Cells*.

[2] Yue F., Li W., Zou J. (2017). Spermidine prolongs lifespan and prevents liver fibrosis and hepatocellular carcinoma by activating MAP1S-mediated autophagy. *Cancer Research*.

[3] Panebianco C., Oben J. A., Vinciguerra M., Pazienza V. (2017). Senescence in hepatic stellate cells as a mechanism of liver fibrosis reversal: a putative synergy between retinoic acid and PPAR-gamma signalings. *Clinical and Experimental Medicine*.

[4] Liu Y. W., Chiu Y. T., Fu S. L., Huang Y. T. (2015). Osthole ameliorates hepatic fibrosis and inhibits hepatic stellate cell activation. *Journal of Biomedical Science*.

[5] Wang C., Zhang F., Cao Y. (2016). Etoposide induces apoptosis in activated human hepatic stellate cells via ER stress. *Scientific Reports*.

[6] Li Y., Chen Y., Huang H. (2017). Autophagy mediated by endoplasmic reticulum stress enhances the caffeine-induced apoptosis of hepatic stellate cells. *International Journal of Molecular Medicine*.

[7] Hernández-Gea V., Hilscher M., Rozenfeld R. (2013). Endoplasmic reticulum stress induces fibrogenic activity in hepatic stellate cells through autophagy. *Journal of Hepatology*.

[8] Chen Y., Liu Z., Liang S. (2008). Role of Kupffer cells in the induction of tolerance of orthotopic liver transplantation in rats. *Liver Transplantation*.

[9] Sica A., Invernizzi P., Mantovani A. (2014). Macrophage plasticity and polarization in liver homeostasis and pathology. *Hepatology*.

[10] Murray P. J., Allen J. E., Biswas S. K. (2014). Macrophage activation and polarization: nomenclature and experimental guidelines. *Immunity*.

[11] Keestra-Gounder A. M., Byndloss M. X., Seyffert N. (2016). NOD1 and NOD2 signalling links ER stress with inflammation. *Nature*.

[12] Xu X., Wang M., Li J. (2018). Tauroursodeoxycholic acid alleviates hepatic ischemia reperfusion injury by suppressing the function of Kupffer cells in mice. *Biomedicine & Pharmacotherapy*.

[13] Guo S., Messmer-Blust A., Wu J. (2014). Role of A20 in cIAP-2 protection against tumor necrosis factor *α* (TNF-*α*)-mediated apoptosis in endothelial cells. *International Journal of Molecular Sciences*.

[14] Ni H.-M., McGill M. R., Chao X., Woolbright B. L., Jaeschke H., Ding W.-X. (2016). Caspase inhibition prevents tumor necrosis factor-*α*–induced apoptosis and promotes necrotic cell death in mouse hepatocytes in vivo and in vitro. *The American Journal of Pathology*.

[15] Wang T., Yang S.-D., Liu S., Wang H., Liu H., Ding W. Y. (2016). 17*β*-estradiol inhibites tumor necrosis factor-*α* induced apoptosis of human nucleus pulposus cells via the PI3K/Akt pathway. *Medical Science Monitor*.

[16] Abdullahi A., Stanojcic M., Parousis A., Patsouris D., Jeschke M. G. (2017). Modeling acute ER stress in vivo and in vitro. *Shock*.

[17] Thompson K. C., Trowern A., Fowell A. (1998). Primary rat and mouse hepatic stellate cells express the macrophage inhibitor cytokine interleukin-10 during the course of activation in vitro. *Hepatology*.

[18] Zhou B., Ling L., Zhang F. (2018). Fibronectin type III domain-containing 5 attenuates liver fibrosis via inhibition of hepatic stellate cell activation. *Cellular Physiology and Biochemistry*.

[19] Lee Y. S., Funk L. H., Lee J. K., Bunge M. B. (2018). Macrophage depletion and Schwann cell transplantation reduce cyst size after rat contusive spinal cord injury. *Neural Regeneration Research*.

[20] Forouzanfar M. H., Afshin A., Alexander L. T. (2015). Global, regional, and national comparative risk assessment of 79 behavioural, environmental and occupational, and metabolic risks or clusters of risks in 188 countries, 1990–2013: a systematic analysis for the Global Burden of Disease Study 2013. *The Lancet*.

[21] Tsochatzis E. A., Bosch J., Burroughs A. K. (2014). Liver cirrhosis. *Lancet*.

[22] Higashi T., Friedman S. L., Hoshida Y. (2017). Hepatic stellate cells as key target in liver fibrosis. *Advanced Drug Delivery Reviews*.

[23] Grabinger T., Bode K. J., Demgenski J. (2017). Inhibitor of apoptosis protein-1 regulates tumor necrosis factor-mediated destruction of intestinal epithelial cells. *Gastroenterology*.

[24] Xu H., Li J., Zhao Y., Liu D. (2017). TNF*α*-induced downregulation of microRNA-186 contributes to apoptosis in rat primary cardiomyocytes. *Immunobiology*.

[25] Kong D., Chen L., Huang W. (2020). Combined therapy with ligustrazine and paeonol mitigates hepatic fibrosis through destroying mitochondrial integrity of stellate cell. *American Journal of Translational Research*.

[26] Li B., Cong M., Zhu Y. (2017). Indole-3-carbinol induces apoptosis of hepatic stellate cells through K63 de-ubiquitination of RIP1 in rats. *Cellular Physiology and Biochemistry*.

[27] Yang J. A., Kong W. H., Sung D. K. (2015). Hyaluronic acid-tumor necrosis factor-related apoptosis-inducing ligand conjugate for targeted treatment of liver fibrosis. *Acta Biomaterialia*.

[28] Hozawa S., Nakamura T., Nakano M. (2008). Induction of matrix metalloproteinase-1 gene transcription by tumour necrosis factor alpha via the p50/p50 homodimer of nuclear factor-kappa B in activated human hepatic stellate cells. *Liver International*.

[29] Zhou J., Huang N., Guo Y. (2019). Combined obeticholic acid and apoptosis inhibitor treatment alleviates liver fibrosis. *Acta Pharmaceutica Sinica B*.

[30] Choi J. S., Jeong I. S., Han J. H., Cheon S. H., Kim S.-W. (2019). IL-10-secreting human MSCs generated by TALEN gene editing ameliorate liver fibrosis through enhanced anti-fibrotic activity. *Biomaterials Science*.

[31] Yao Y., Wang Y., Zhang Z. (2016). Chop deficiency protects mice against bleomycin-induced pulmonary fibrosis by attenuating M2 macrophage production. *Molecular Therapy*.

[32] Yang Y., Wu X. Q., Li W. X. (2018). PSTPIP2 connects DNA methylation to macrophage polarization in CCL4-induced mouse model of hepatic fibrosis. *Oncogene*.

[33] Larson-Casey J. L., Deshane J. S., Ryan A. J., Thannickal V. J., Carter A. B. (2016). Macrophage Akt1 kinase-mediated mitophagy modulates apoptosis resistance and pulmonary fibrosis. *Immunity*.

[34] Xie Y., Wen H., Yan K. (2016). Toxoplasma gondii GRA15 effector-induced M1 cells ameliorate liver fibrosis in mice infected with Schistosomiasis japonica. *Cellular & Molecular Immunology*.

[35] Yuan D., Huang S., Berger E. (2017). Kupffer cell-derived Tnf triggers cholangiocellular tumorigenesis through JNK due to chronic mitochondrial dysfunction and ROS. *Cancer Cell*.

